# Delineamento e Racional do Estudo Rosa dos Ventos: Estudo Multicêntrico do Tipo Coorte de Pacientes com Insuficiência Cardíaca com Fração de Ejeção Reduzida ou Moderadamente Reduzida no Brasil

**DOI:** 10.36660/abc.20240120

**Published:** 2024-11-07

**Authors:** Dhayn Cassi de Almeida Freitas, Larissa Maria de Paula Rebouças da Costa, Wilson Nadruz, Fabiana G. Marcondes-Braga, Jefferson Luis Vieira, Sabrina Bernardez-Pereira, Wilson Rodrigues Barbosa, Silvia Marinho Martins Alves, Gabriela Arcoverde Wanderley, Camila Nogueira Leandro Lira, Lucas Yugi de Souza Terui, Ana Luísa Guedes de França e Silva, Alana de Oliveira Castro, Aguinaldo F. Freitas, José Albuquerque de Figueiredo, Renato D. Lopes, Miguel Morita Fernandes-Silva, Odilson Marcos Silvestre

**Affiliations:** 1 Universidade Federal do Acre Rio Branco AC Brasil Universidade Federal do Acre, Rio Branco, AC – Brasil; 2 Universidade Estadual de Campinas Campinas SP Brasil Universidade Estadual de Campinas, Campinas, SP – Brasil; 3 Hospital das Clínicas da Faculdade de Medicina da Universidade de São Paulo Instituto do Coração São Paulo SP Brasil Instituto do Coração – Hospital das Clínicas da Faculdade de Medicina da Universidade de São Paulo, São Paulo, SP – Brasil; 4 Hospital de Messejana Dr. Carlos Alberto Studart Unidade de Insuficiência Cardíaca Fortaleza CE Brasil Unidade de Insuficiência Cardíaca – Hospital de Messejana Dr. Carlos Alberto Studart, Fortaleza, CE – Brasil; 5 Faculdade de Medicina da Universidade Federal do Ceará Fortaleza CE Brasil Programa de Pós-Graduação em Ciências Cardiovasculares – Faculdade de Medicina da Universidade Federal do Ceará, Fortaleza, CE – Brasil; 6 Hospital Israelita Albert Einstein São Paulo SP Brasil Hospital Israelita Albert Einstein, São Paulo, SP – Brasil; 7 Clínica Silvestre Santé Rio Branco AC Brasil Clínica Silvestre Santé, Rio Branco, AC – Brasil; 8 Pronto-Socorro Cardiológico Universitário de Pernambuco Prof. Luiz Tavares Recife PE Brasil Pronto-Socorro Cardiológico Universitário de Pernambuco Prof. Luiz Tavares, Recife, PE – Brasil; 9 Universidade de Pernambuco Recife PE Brasil Universidade de Pernambuco, Recife, PE – Brasil; 10 Universidade Federal de Goiás Goiânia GO Brasil Universidade Federal de Goiás, Goiânia, GO – Brasil; 11 Universidade Federal do Maranhão São Luís MA Brasil Universidade Federal do Maranhão, São Luís, MA – Brasil; 12 Duke University Medical Center Durham NC EUA Duke University Medical Center, Durham, NC – EUA; 13 Brazilian Clinical Research Institute São Paulo SP Brasil Brazilian Clinical Research Institute (BCRI), São Paulo, SP – Brasil; 14 Universidade Federal do Paraná Curitiba PR Brasil Universidade Federal do Paraná, Curitiba, PR – Brasil

**Keywords:** Insuficiência Cardíaca, Brazil, Estratégias de Saúde, Prognosis

## Abstract

**Fundamento:**

O Brasil é um país com diferentes biomas e desigualdades sociais. Existem poucos dados disponíveis sobre as diferenças regionais e o prognóstico da insuficiência cardíaca (IC) no país.

**Objetivo:**

O estudo Rosa dos Ventos tem como objetivo investigar as diferenças regionais e o prognóstico atual de pacientes com IC com fração de ejeção reduzida ou moderadamente reduzida no Brasil.

**Métodos:**

Este é um estudo prospectivo, multicêntrico, observacional, do tipo coorte que incluirá pacientes ambulatoriais com idade superior a 18 anos com IC e fração de ejeção < 50% em 30 centros privados distribuídos nas regiões brasileiras. Um total de 2500 pacientes serão incluídos entre junho de 2021 e outubro de 2023, com um período de 12 meses de seguimento. Coletaremos dados sobre status clínico e socioeconômico, prescrição médica e resultados de exames cardiológicos. Serão realizados telefonemas para o seguimento dos pacientes seis e 12 meses após a inclusão para coleta de informações sobre visitas ao departamento de emergência, internações e mortalidade.

**Conclusão:**

O estudo Rosa dos Ventos permitirá uma caracterização mais precisa da IC crônica no Brasil. Essa iniciativa proverá informações relevantes para o desenvolvimento de estratégias de manejo efetivas para mitigar o impacto dessa condição sobre os pacientes e o sistema de saúde.

## Graphical abstract

**Figure:**
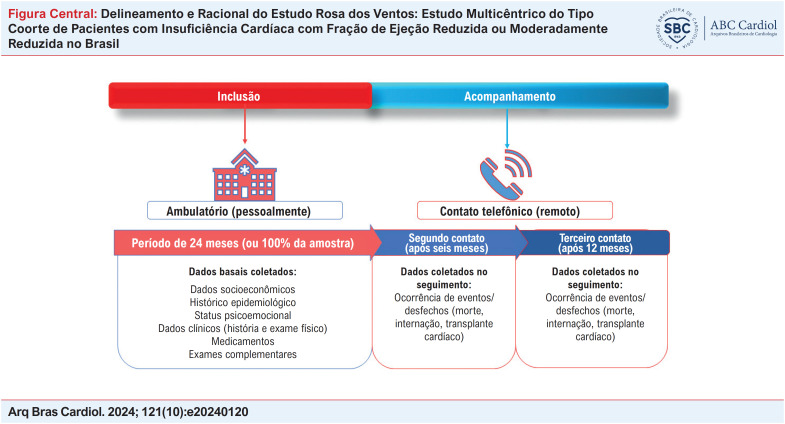
Fluxograma do estudo.

## Introdução

A insuficiência cardíaca (IC) afeta aproximadamente 64 milhões de pessoas em todo o mundo, com cerca de quase dois milhões de casos relatados no Brail.^1,2^ A IC está associada à elevada mortalidade, morbidade, internações recorrentes e baixa qualidade de vida. Entre 2008 e 2019, a IC foi responsável por cerca de três milhões de internações hospitalares no Brasil, e quase 2,7 trilhões de dólares ao sistema público de saúde.^3-6^

O Brasil é dividido em cinco regiões geográficas; as regiões norte e nordeste apresentam elevadas taxas de pobreza, 15,0% e 16,5% respectivamente, em contraste com as regiões sul e sudeste, que apresentam taxas significativamente mais baixas de 3% e 4%, respectivamente.^7^ O acesso a condições básicas de moradia e de serviços de saúde é notavelmente reduzido nas regiões norte e nordeste, em contraste a outras áreas.^6^ Ainda, as regiões norte e nordeste encaram o desafio de várias doenças tropicais negligenciadas. Essas incluem malária, que afeta 140 mil indivíduos anualmente,^8^ e as arboviroses como dengue e chikungunya, que resulta em mais de 380,000 casos por ano, principalmente durante a época de chuva.^9^ Além disso, essas regiões foram responsáveis por 97,05% dos casos de doença de Chagas no ano 2020.^10^ A distribuição heterogênea de pobreza, desigualdade social, dimensões geográficas extensas, e a prevalência de doenças tropicais negligenciadas impõe grandes dificuldades em prover assistência em saúde abrangente e eficiente. Esses desafios levam a enormes disparidades na saúde,^7,11^ que poderiam influenciar a epidemiologia da IC.

Dada a complexa natureza do tratamento da IC e as questões relacionadas à saúde mencionadas, o manejo da IC torna-se ainda mais complexo. Compreender as causas, o tratamento e o prognóstico da IC no Brasil, bem como as variações regionais é de grande importância para direcionais estratégias de saúde pública e aliviar a carga dessa doença.^12-14^ Estudos anteriores realizados no Brasil tentaram determinar características clínicas e mortalidade de pacientes com IC crônica.^15^ Contudo, esses esforços têm se limitado a centros únicos de referência ou dependido de dados administrativos que carecem tanto de informação detalhada como de acurácia. Até o momento, não há nenhum estudo multicêntrico caracterizando, de maneira abrangente, a IC crônica no Brasil – um país de renda média com desigualdades socioeconômicas marcantes e dimensões geográficas continentais. Portanto, delineamos um estudo multicêntrico para avaliar as características clínicas, o tratamento, e o prognóstico da IC no Brasil, considerando as diferenças regionais geográficas. O objetivo deste estudo é descrever as características clínicas e epidemiológicas desses pacientes, traçar as especificidades das abordagens terapêuticas, e avaliar os fatores que afetam seu prognóstico.

## Métodos

### Delineamento do estudo

**Figura 1:**
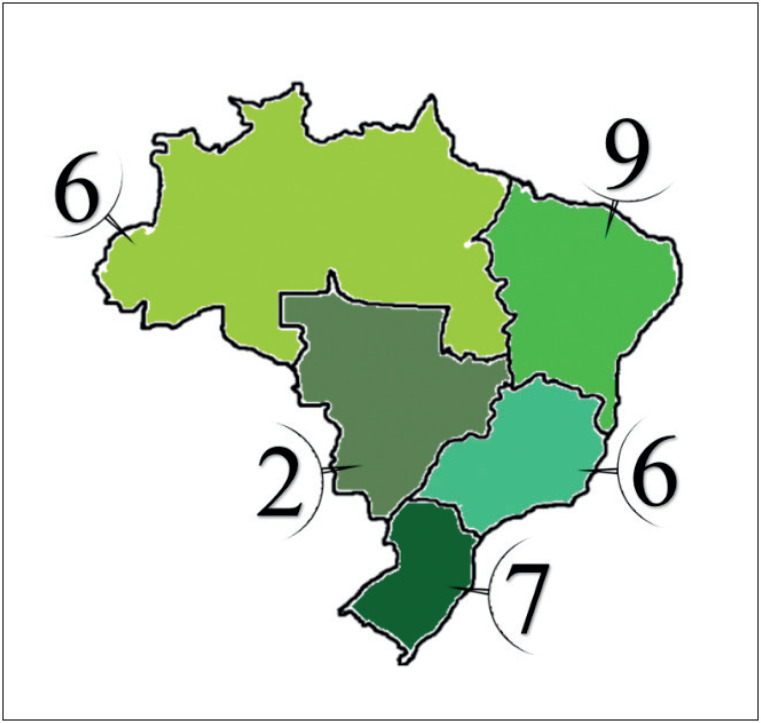
Número de centros participantes no Estudo Rosa dos Ventos por região geográfica no Brasil.

O estudo Rosa dos Ventos é um estudo prospectivo, multicêntrico do tipo coorte para avaliar os pacientes diagnosticados com IC crônica e fração de ejeção (FE) reduzida (ICFEr) ou moderadamente reduzida (ICFEmr) no Brasil. Essa coorte incluiu 2500 pacientes ambulatoriais de 30 centros localizados em 23 unidades federativas brasileiras (Figura 1).

### Local do estudo e tamanho amostral

Calculamos um tamanho amostral para detectar uma diferença de 10% na incidência cumulativa do desfecho composto por morte e internação hospitalar em um ano de seguimento, comparando-se especificamente duas das cinco regiões. Com base em um estudo prévio de um centro de IC no Brasil,^11^ assumimos uma incidência acumulada de 17,7% para a região com o risco mais baixo. Estimamos que seria necessário um mínimo de 466 participantes por região para detectar uma diferença de 10%, com um poder de 80%, aplicando um valor de p=0,05 bilateral. Esse valor de p foi escolhido para acomodar múltiplas comparações entre as cinco regiões, aplicando-se o ajuste de Bonferroni e um nível de significância de 5%. Considerando-se uma perda potencial de 5% durante o seguimento, e um desequilíbrio no tamanho amostral entre as regiões, estabelecemos um tamanho amostral de 2500 participantes.

O estudo foi iniciado em junho de 2021, e a fase de recrutamento foi antecipada para ser concluída antes de outubro de 2023. Os pacientes foram recrutados em ambulatórios de 30 locais participantes. Planeja-se concluir o seguimento de 12 meses de todos os pacientes até novembro de 2024.

### População

Nós incluiremos indivíduos com um diagnóstico de IC crônica independentemente da etiologia, e que estejam recebendo tratamento ambulatorial.

Os critérios de inclusão são idade igual ou superior a 18 anos, e um ecocardiograma realizado nos últimos 12 meses, mostrando uma FE do ventrículo esquerdo (FEVE) abaixo de 50%. Serão excluídos indivíduos pertencentes a populações indígenas, por questões éticas, e indivíduos com déficit cognitivo que afetaria sua capacidade de compreender e responder as perguntas do questionário. Os critérios de elegibilidade estão resumidos na Tabela 1.

**Tabela 1:** Critérios de elegibilidade dos pacientes

Inclusão	Exclusão
Idade ≥ 18 anos	População indígena
FEVE < 50%*	Déficit cognitivo

FEVE: fração de ejeção do ventrículo esquerdo;

* ecocardiograma realizado nos últimos 12 meses.

### Consentimento informado

Os participantes serão avaliados no ambulatório de um dos centros participantes. Um pesquisador será responsável por explicar o estudo e apresentar o termo de consentimento a cada paciente. Se o paciente concordar em participar, ele assinará duas vias do termo, uma ficará com o pesquisador, e a outra será entregue ao participante.

Para participantes analfabetos ou aqueles com limitações para ler e compreender o termo, uma pessoa designada fará a leitura do termo para eles. Para esses, será empregado um termo diferente, em que o paciente indicará concordância em participar por sua impressão digital. Para validar esse processo, duas testemunhas estarão presentes: a pessoa responsável e uma terceira pessoa imparcial sem qualquer afiliação com o estudo ou com a equipe de pesquisa. Ambas as testemunhas assinarão o termo.

### Linha do tempo do participante

Cada paciente será contatado por telefone seis e 12 meses após a inclusão no estudo por um dos investigadores do estudo. Para minimizar o risco de perda no seguimento, coletaremos o número de telefone do paciente, seu endereço residencial, e informações de contato de pelo menos dois parentes ou amigos próximos durante a coleta de dados basais. Essa informação será atualizada durante os seis meses de contato telefônico. A Figura Central resume o fluxograma deste estudo.

### Coleta, manejo e análise dos dados

#### Coleta de dados

Dados basais e de seguimento serão coletados usando um Formulário de Relato de Caso (FRC). O médico atendente conduzirá o exame clínico, e um investigador registrará as informações relevantes. Além disso, nós registraremos detalhes da prescrição médica, bem como os resultados do ecocardiograma, do eletrocardiograma, e testes laboratoriais.

A coleta de dados será conduzida exclusivamente por um FRC eletrônico integrado à plataforma de dados RedCap (https://redcapbrasil.com.br/). Todos os centros participantes terão acesso ao FRC, permitindo-lhes incluir participantes e assegurar completa confiabilidade do instrumento e dos dados dos pacientes incluídos.

Os seguintes dados basais serão coletados:

– Dados demográficos e socioeconômicos: data de nascimento, sexo, etnia, número de telefone, endereço residencial, estado civil, escolaridade, profissão, renda per capita;
– Outros indicadores socioeconômicos: condições de moradia, condições sanitárias básicas, exposição a áreas verdes, exposição a enchentes, acesso à Internet, compra de medicamentos;
– História clínica prévia: fatores de risco cardiovascular, eventos cardiovasculares prévios, comorbidades, depressão, consumo de álcool e tabagismo, história de doenças tropicais negligenciadas, incluindo COVID-19;
– Sinais e sintomas: classe funcional (*New York Heart Association*), ortopneia, peso, altura, pressão arterial, frequência cardíaca, distensão da veia jugular com estimativa da pressão da veia jugular, S3, edema periférico, ascite e/ou hepatomegalia, classificação de perfusão e sons respiratórios;
– Dados do ecocardiograma: presença de doença cardíaca valvar, presença de válvula prostética, FEVE, diâmetro anteroposterior do átrio esquerdo, medida do Ventrículo Direito (VD), massa do Ventrículo Esquerdo (VE), massa do VE indexada pela superfície corporal, diâmetro diastólico e sistólico do VE, espessura da parede do VE, função diastólica do VE, função sistólica do VD, e presença de alteração segmental do VE;
– Dados eletrocardiográficos: ritmo, distúrbios de condição, duração do complexo QRS, duração do intervalo QT, intervalo QT corrigido;
– Dados laboratoriais: células sanguíneas, eletrólitos, lipídios, proteínas, hormônios tireoidianos, troponina, e porção N-terminal do pró-hormônio do peptídeo natriurético do tipo B (NT-proBNP);
– Tratamento médico da IC com base em diretriz: betabloqueadores específicos para IC, inibidor da enzima conversora de angiotensina, bloqueador de receptor de angiotensina, sacubitril-valsartana, antagonista de mineralocorticoide, hidralazina, nitrato, inibidor de SGLT2.
– Outros medicamentos: diurético de alça, diurético tiazídico, digoxina, amiodarona, bloqueador de canal de cálcio, anticoagulante, agonista de GLP1, antidiabéticos orais, insulina, agentes antiplaquetários, e estatina.

Durante as visitas de acompanhamento, serão coletados dados sobre status vitais, transplante cardíaco, internações hospitalares, e visitas ao departamento de emergência. A data de cada evento e o número de internações hospitalares ou visitas ao departamento de emergência também serão registrados. Para indivíduos que forem a óbito, a causa do óbito será obtida de prontuários médicos, pessoas próximas, e certificado de óbito.

#### Manejo dos dados

Para assegurar a integridade e a acurácia dos dados coletados, foi implementado um plano robusto de manejo de dados. Esse incluiu a formulação de um protocolo abrangente de coleta de dados, procedimentos padrões de entrada de dados, e o estabelecimento de um sistema de armazenamento seguro de dados. Os dados de cada participante foram cuidadosamente registrados, verificados, e armazenados utilizando-se os instrumentos avançados de captura de dados eletrônicos pelo sistema RedCap^®^.

Medidas precisas de confidencialidade foram implementadas para proteger a privacidade e a anonimidade dos participantes do estudo. Verificações regulares e minuciosas de qualidade e auditorias foram conduzidas pela *The Brazilian Clinical Research Institute* (BCRI) para identificar quaisquer discrepâncias ou inconsistências nos dados. A *The Brazilian Clinical Research Institute* (BCRI) prioriza práticas de manejo de dados que seguem diretrizes éticas e regulatórias, assegurando a confiabilidade e a validade dos achados do estudo.

### Análise estatística

Os dados categóricos serão descritos como frequências e proporções. As variáveis contínuas serão avaliadas quanto à distribuição gaussiana, examinando-se a forma, assimetria, curtose e, se necessário, conduzir o teste de Kolmogorov-Smirnov. Se os dados seguirem uma distribuição normal, serão apresentados como média e desvio padrão; caso contrário, como mediana (percentil 25 e percentil 75).

As características basais serão comparadas entre diferentes macrorregiões brasileiras usando o teste do qui-quadrado para variáveis categóricas, o teste ANOVA unidirecional para variáveis contínuas com distribuição normal e o teste de Kruskal-Wallis para variáveis contínuas sem distribuição normal.

Quando os testes indicarem uma diferença estatisticamente significativa entre os cinco grupos, continuaremos as análises com comparações em pares. Para controlar um aumento potencial na taxa de erro tipo I devido a múltiplas comparações, aplicaremos a correção de Bonferroni aos nossos testes estatísticos. Por outro lado, se os testes globais não rejeitarem a hipótese nula, indicando ausência de diferença significativa entre os cinco grupos, nenhuma outra comparação em pares será conduzida.

Associações entre as variáveis e dos desfechos serão analisadas por curvas de Kaplan-Meier e modelos de regressão Cox, com ajustes para potenciais fatores de confusão. A análise estatística será realizada com o programa Stata versão 14.0, e será adotado um nível de significância (*α*) de 0,05 (p<0,05).

### Aprovação ética

O estudo Rosa dos Ventos obteve aprovação ética do Comitê de ética da Universidade Federal do Acre (CAAE: 25756919.9.1001.5010), bem como de cada comitê de ética local, de acordo com os regulamentos de cada estado participante.

### Estrutura organizacional

#### Supervisão

Este estudo Rosa dos Ventos é uma iniciativa acadêmica, em colaboração com vários centros no Brasil. O comitê executivo, juntamente com os times operacionais, fará a supervisão dos aspectos científicos e operacionais do estudo. Membros do comitê coordenador e do comitê executivo serão responsáveis pelo relato dos resultados.

## Discussão

O estudo Rosa dos Ventos será o primeiro registro grande e abrangente de pacientes com ICFEr e ICFEmr no Brasil. Dadas suas dimensões continentais, com a quinta maior população do mundo, o Brasil é caracterizado por suas disparidades socioeconômicas que influenciam a incidência, o prognóstico, e a distribuição das doenças cardiovasculares. Esse registro da IC tem como objetivo apresentar insights valiosos ao comportamento da doença em um país de renda média, incluindo diferenças regionais e socioeconômicas, bem como IC resultante de doenças tropicais negligenciadas como a doença de Chagas.

Um estudo conduzido por Yusuf et al.^16^ revelou taxas mais altas de mortalidade por doença cardiovascular em países de renda baixa e média em comparação a países de alta renda (3,99 eventos por 1000 pessoas-ano *versus* 5,38 e 6,43 eventos per 1000 pessoas-ano, respectivamente; p<0,001).^16,17^ Esse achado sugere que possam existir padrões similares ao se comparar regiões economicamente distintas no Brasil. Além disso, já foi demonstrada uma forte associação entre uma prevalência mais alta de fatores de risco não controlados, comumente observada em países não desenvolvidos, e piores desfechos cardiovasculares, incluindo uma maior incidência de IC.^16,18^ Portanto, podemos esperar uma relação similar no país.

Determinantes socioeconômicos influenciam significativamente as características e o prognóstico da ICFEmr. No entanto, é importante notar que dados disponíveis sobre IC que influenciam as estratégias de manejo no Brasil derivam, primariamente, de registros americanos e europeus, podem não representar, de maneira precisa, a população de pacientes com IC em países em desenvolvimento. Ainda, ensaios clínicos randomizados conduzidos em centros terciários especializados poderiam aumentar a prevalência de etiologias específicas.^14,19,20^ Embora a doença cardíaca seja a principal causa de IC em países desenvolvidos, antecipamos que a hipertensão e a doença de Chagas terão um impacto significativo como causas de IC no Brasil. Além disso, esperamos que a adesão a terapias guiadas por diretrizes seja menor que a relatada em países europeus e americanos.^5,14,17^

Para implementar estratégias eficazes no manejo da IC, é essencial ter um entendimento detalhado de como a doença se apresenta. Este será um estudo pioneiro conduzindo uma investigação multicêntrica de pacientes com IC crônica em diferentes regiões brasileiras. A premissa é que essa informação aumentará nossa compreensão sobre a manifestação da doença, comorbidades, e padrões de tratamento. Ao identificar disparidades regionais, esperamos desenvolver novas estratégias lidar com a doença e apoiar políticas públicas em saúde futuras ajustadas às características peculiares de cada região brasileira.^19-23^

O estudo tem limitações. É possível que exista potencial viés de informação oriundo de dados relatados pelos próprios pacientes. Contudo, o estudo segue protocolos padronizados e mantém esforços de formação continuada para minimizar vieses em geral.

Em conclusão, o estudo Rosa dos Ventos tem como objetivo caracterizar variações regionais na IC e avaliar o prognóstico da doença em 12 meses no Brasil. Os resultados fornecerão *insights* valiosos às variações na prevalência de IC, práticas de manejo, e desfechos dos pacientes em diferentes regiões do país. Os resultados servirão como base para intervenções e alocação de recursos, assegurando que regiões com maiores desigualdades recebam o apoio e os recursos necessários para melhorar os desfechos dos pacientes e reduzir a desigualdade na assistência em saúde. Ainda, um estudo com um registro mais abrangente provavelmente ajudará a direcionar o desenvolvimento de diretrizes baseadas em evidência e melhores práticas que abordem as necessidades específicas de diferentes regiões, fomentando abordagens mais igualitárias e mais bem adaptadas ao manejo da IC em todo o país.
