# Supplementary material for: Delineamento e Racional do Estudo Rosa dos Ventos: Estudo Multicêntrico do Tipo Coorte de Pacientes com Insuficiência Cardíaca com Fração de Ejeção Reduzida ou Moderadamente Reduzida no Brasil
Source: Arq Bras Cardiol. 2024 Nov 7;121(10):e20240120. [Article in Portuguese] doi: 10.36660/abc.20240120 (PMC11634221; doi:10.36660/abc.20240120)
Supplement: Translation

# Translation: Rosa dos Ventos Multicenter Cohort Study of Patients with Reduced or Mildly Reduced Ejection Fraction Heart Failure in Brazil: Rationale and Design

**DOI:** 10.36660/abc.20240120i

**Authors:** Dhayn Cassi de Almeida Freitas, Larissa Maria de Paula Rebouças da Costa, Wilson Nadruz, Fabiana G. Marcondes-Braga, Jefferson Luis Vieira, Sabrina Bernardez-Pereira, Wilson Rodrigues Barbosa, Silvia Marinho Martins Alves, Gabriela Arcoverde Wanderley, Camila Nogueira Leandro Lira, Lucas Yugi de Souza Terui, Ana Luísa Guedes de França e Silva, Alana de Oliveira Castro, Aguinaldo F. Freitas, José Albuquerque de Figueiredo, Renato D. Lopes, Miguel Morita Fernandes-Silva, Odilson Marcos Silvestre

**Affiliations:** 1 Rio Branco AC Brazil Universidade Federal do Acre, Rio Branco, AC – Brazil; 2 Campinas SP Brazil Universidade Estadual de Campinas, Campinas, SP – Brazil; 3 São Paulo SP Brazil Instituto do Coração – Hospital das Clínicas da Faculdade de Medicina da Universidade de São Paulo, São Paulo, SP – Brazil; 4 Fortaleza CE Brazil Unidade de Insuficiência Cardíaca – Hospital de Messejana Dr. Carlos Alberto Studart, Fortaleza, CE – Brazil; 5 Fortaleza CE Brazil Programa de Pós-Graduação em Ciências Cardiovasculares – Faculdade de Medicina da Universidade Federal do Ceará, Fortaleza, CE – Brazil; 6 São Paulo SP Brazil Hospital Israelita Albert Einstein, São Paulo, SP – Brazil; 7 Rio Branco AC Brazil Clínica Silvestre Santé, Rio Branco, AC – Brazil; 8 Recife PE Brazil Pronto-Socorro Cardiológico Universitário de Pernambuco Prof. Luiz Tavares, Recife, PE – Brazil; 9 Recife PE Brazil Universidade de Pernambuco, Recife, PE – Brazil; 10 Goiânia GO Brazil Universidade Federal de Goiás, Goiânia, GO – Brazil; 11 São Luís MA Brazil Universidade Federal do Maranhão, São Luís, MA – Brazil; 12 Durham NC USA Duke University Medical Center, Durham, NC – USA; 13 São Paulo SP Brazil Brazilian Clinical Research Institute (BCRI), São Paulo, SP – Brazil; 14 Curitiba PR Brazil Universidade Federal do Paraná, Curitiba, PR – Brazil

**Keywords:** Heart failure, Brasil, Health Strategies, Prognosis

## Abstract

**Background:**

Brazil is a country with different biomes and social disparities. There are limited data available on regional differences and prognosis of heart failure (HF) in the country.

**Objective:**

The Rosa dos Ventos study aims to investigate regional differences and the current prognosis of HF outpatients with reduced or mildly reduced ejection fraction in Brazil.

**Methods:**

This is a prospective, multicenter, observational cohort study that will include outpatients older than 18 years with HF and an ejection fraction < 50% in 30 public and private centers distributed in all Brazilian regions. A total of 2,500 patients will be enrolled from June 2021 and October 2023, with a 12-month follow-up period. We will collect data on socioeconomic and clinical status, medical prescription and results of cardiology tests. Follow-up phone calls will be made at 6 and 12 months after inclusion to collect information regarding emergency room visits, hospitalization and mortality.

**Conclusion:**

The Rosa dos Ventos study will allow a more accurate characterization of chronic HF in Brazil. This initiative will provide relevant information for the development of effective management strategies to mitigate the impact of this condition on patients and the healthcare system.

## Introduction

Heart failure (HF) affects approximately 64 million people worldwide, with nearly two million cases reported in Brazil.^1,2^ HF is associated with high mortality, morbidity, recurrent hospitalizations, and poor quality of life. From 2008 to 2019, HF accounted for approximately three million hospitalizations in Brazil, and nearly 2.7 trillion dollars for the public healthcare system.^3-6^

Brazil is divided into five geographical regions; the North and Northeast regions show elevated poverty rates of 15.0% and 16.5%, respectively, in contrast to the South and Southeast regions, which experience significantly lower rates of 3% and 4%, respectively.^7^ Access to basic housing conditions and healthcare services are notably diminished in the North and Northeast regions, in contrast to other areas.^6^ Furthermore, the North and Northeast regions face the challenge of various tropical neglected diseases. These include malaria, affecting 140,000 individuals annually,^8^ along with arboviruses like dengue and chikungunya, which result in over 380,000 cases per year, primarily during the rainy season.^9^ Additionally, these regions accounted for 97.05% of Chagas disease cases in the year of 2020.^10^ The heterogeneous distribution of poverty, social inequality, extensive geographic dimensions, and the prevalence of neglected tropical diseases pose considerable hurdles in delivering comprehensive and efficient healthcare. These challenges lead to notable health disparities,^7,11^ which could influence the epidemiology of HF.

Given the complex nature of HF treatment and the aforementioned public health-related issues, the management of HF becomes even more intricate. Understanding the causes, treatment, and prognosis of HF in Brazil, as well as recognizing regional variations, holds paramount importance for guiding public health strategies and alleviating the burden of this disease.^12-14^ Previous studies performed in Brazil have attempted to characterize clinical characteristics and mortality of patients with chronic HF.^15^ However, these efforts have been limited to single referral centers or have been reliant on administrative data that lack both detailed information and accuracy. To date, there is no multicenter study that comprehensively characterizes chronic HF in Brazil – a middle-income country with marked socioeconomic inequalities and continental geographical dimensions. Therefore, we have designed a multicenter cohort study to evaluate the clinical characteristics, treatment, and prognosis of HF in Brazil, considering the geographical regional differences. This study aims to describe the epidemiological and clinical characteristics of these patients, outline the specifics of their treatment approaches, and assess the factors affecting their prognosis.

## Methods

### Study design

The Rosa dos Ventos Study is a prospective and multicenter cohort study to evaluate patients diagnosed with chronic HF with reduced (HFrEF) or midly reduced ejection fraction (HFmrEF) in Brazil. This cohort included 2,500 outpatients distributed across 30 centers located in 23 Brazilian federative units (Figure 1).

**Figure 1:** Number of participating centers in the Rosa dos Ventos Study by Brazilian geographic regions.

### Study setting and sample size

We performed a sample size calculation to detect a 10% difference in the cumulative incidence of the composite endpoint of death and hospitalization in one-year follow-up period, specifically comparing two out of the five regions. Based on a previous report from a Brazilian HF center,^11^ we assumed a one-year cumulative incidence of 17.7% for the region with the lowest risk. We estimated that a minimum of 466 participants per region would be required to detect a 10% risk-difference with a power of 80% using a two-sided p=0.05. This p-value was chosen to accommodate multiple comparisons across the five regions, employing Bonferroni adjustment with a 5% significance level. Taking into consideration a potential 5% loss to follow-up and uneven sample sizes across regions, we established a total sample size of 2500 participants.

The study was initiated in June 2021, and the recruitment phase was anticipated to conclude by October 2023. Patient enrollment was conducted within outpatient services across 30 participating locations. The 12-month follow-up for all patients is planned to be finalized by November 2024.

### Population

We will include individuals who have been diagnosed with chronic HF irrespective of etiology and are currently receiving outpatient care.

Inclusion criteria require age greater than or equal to 18 years, and an echocardiogram performed within the previous 12 months, showing left ventricle ejection fraction (LVEF) below 50%.

Exclusions will be made for individuals belonging to the indigenous population, due to ethical reasons, or those with cognitive impairment that could hinder their ability to understand and respond to interview questions. The eligibility criteria are summarized in Table 1.

**Table 1:** Eligibility criteria

Inclusion	Exclusion
Age ≥ 18 years	Indigenous population
LVFE < 50%*	Presence of cognitive impairment

LVEF: Left ventricular ejection fraction;

* echocardiogram performed within the previous 12 months.

### Informed consent

Participants will be approached within the outpatient service in one of the participating centers. A researcher will be responsible to explain the study and present the informed consent form to each patient, who will sign two copies of the consent form if he/she agrees to participate. One copy will be retained by the researcher, while the other will be given to the participant.

For illiterate participants or those with limitations to read and understand the form, a designated responsible person will read out the informed consent to them. For these patients, a different form will be employed, in which the patient will indicate his/her agreement through a fingerprint signature. To validate this process, two witnesses will be present: the responsible person and an impartial third party without any affiliation with the study or research team. Both witnesses will also provide their signatures on the form.

### Participant timeline

Each patient will be contacted by phone at six and 12 months after enrollment by one of the study investigators. To minimize the risk of follow-up loss, we will collect patient's phone contact, residential address, and the contact information of at least two relatives or close friends during baseline data collection. This information will be updated during the six-month phone contact. Central Illustration summarizes the flowchart of this study.

### Data collection, management, and analysis

#### Data collection

Baseline and follow-up data will be collected utilizing a case report form (CRF). The attending physician will conduct the clinical examination, while as investigator will record the relevant information. Additionally, we will record details from the medical prescription, as well as the results of the echocardiogram, electrocardiogram, and laboratory tests.

The data collection will be conducted exclusively via an electronic CRF integrated into the RedCap data platform (https://redcapbrasil.com.br/). All participant centers will have access to the CRF, enabling them to include participants while ensuring the complete confidentiality of the instrument and the data of the enrolled patients.

The following baseline data will be collected:

– Demographic and socioeconomic data: date of birth, sex, ethnicity, telephone number, residential address, marital status, education level, occupation, per capita income;
– Other socioeconomic indicators: housing conditions, basic sanitation conditions, exposure to green areas, exposure to flooding, internet access, purchase of medicines;
– Previous medical history: cardiovascular risk factors, previous cardiovascular events, comorbidities, depression, alcohol consumption and smoking, history of neglected tropical diseases and other infections, including COVID-19;
– Signs and symptoms: functional class (New York Heart Association), orthopnea, weight, height, blood pressure, heart rate, jugular distension with estimative of jugular venous pressure, S3, peripheral edema, ascites and/or hepatomegaly, classification of rales and perfusion;
– Echocardiogram data: presence of valvar heart disease, presence of prosthetic valve, LVEF, anteroposterior diameter of the left atrium, right ventricle (RV) measurement, left ventricular (LV) mass, LV mass indexed by body surface, LV diastolic and systolic diameters, LV wall thickness, LV diastolic function, RV systolic function, presence of LV segmental alterations;
– ECG data: rhythm, conduction disturbances, QRS complex duration, QT interval duration, corrected QT interval;
– Laboratory test data: blood cells, electrolytes, lipids, proteins, thyroid hormones, troponin, and N-terminal prohormone of brain natriuretic peptide (NT-proBNP);
– HF guideline-directed medical treatment: HF-specific beta-blocker, angiotensin-converting enzyme inhibitor, angiotensin receptor blocker, sacubitril-valsartan, mineralocorticoid antagonist, hydralazine, nitrate, SGLT2 inhibitor;
– Other medications: loop diuretic, thiazide diuretic, digoxin, amiodarone, calcium channel blocker, anticoagulant, GLP1 agonist, oral antidiabetics, insulin, antiplatelet agents, and statin.

During follow-up visits, data on vital status, heart transplantation, hospitalizations, and visits to the emergency department will be collected. The date of each event and the number of hospitalizations or emergency department visits will also be recorded. For deceased individuals, the cause and date of death will be obtained from medical records, next of kin, and death certificate.

#### Data management

To ensure the integrity and accuracy of the collected data, a robust data management plan has been implemented. This included the formulation of a comprehensive data collection protocol, standardized procedures for data entry, and the establishment of a secure storage system. Each participant's data were carefully recorded, verified, and securely stored using advanced electronic data capture tools via RedCap^®^ system.

Stringent confidentiality measures were enacted to safeguard the privacy and anonymity of the study participants. Regular and thorough quality checks and audits were conducted by Brazilian Clinical Research Institute (BCRI) to identify any discrepancies or inconsistencies in the data. The Brazilian Clinical Research Institute (BCRI) prioritizes data management practices that adhere to ethical guidelines and regulatory requirements, ensuring the reliability and validity of the study findings.

### Statistical analysis

Categorical data will be described as frequencies and proportions. Continuous variables will be assessed for Gaussian distribution by examining the shape of the distribution, skewness, kurtosis, and, if necessary, conducting the Kolmogorov-Smirnov test. If the data follow a normal distribution, they will be presented as mean ± standard deviation; otherwise, as median (25th percentile, 75th percentile).

Baseline characteristics will be compared among different macro-regions in Brazil using the chi-squared test for categorical variables, one-way ANOVA for normally distributed continuous variables, and the Kruskal-Wallis's test for non-normally distributed continuous variables.

When the tests indicate a significant difference between the five groups, we will proceed with pairwise comparisons. To control for potential increase in Type I error rate due to multiple comparisons, we will apply the Bonferroni correction to our statistical tests. Conversely, if the global tests do not reject the null hypothesis, indicating no significant difference between the five groups, no further pairwise comparisons will be conducted.

Associations between variables and outcomes will be analyzed using Kaplan-Meier curves and Cox regression models, with adjustments for potential confounders. The statistical analysis will be performed with Stata version 14.0, and a significance level (α) of 0.05 (p<0.05) will be adopted.

### Ethics approval

The Rosa dos Ventos study has obtained ethical approval from the Ethics Committee of the Universidade Federal do Acre (CAAE: 25756919.9.1001.5010), as well as from each local institutional ethics board, in accordance with the regulations of each participating state.

### Organizational structure

#### Supervision

This Rosa dos Ventos study is an academically led initiative in collaboration with several centers in Brazil. The executive committee, together with the operational teams will oversee the scientific and operational aspects of the study. The executive and steering committee members will be responsible for the reporting of the results.

## Discussion

The Rosa dos Ventos Study will be the first large and most comprehensive registry of patients with HFrEF and HFmrEF in Brazil. Given its continental dimensions, with the fifth largest population in the world, Brazil is characterized by socioeconomic disparities that influence the incidence, prognosis, and distribution of cardiovascular diseases. This HF registry aims to provide valuable insights into the behavior of the disease in a middle-income country, including regional and socioeconomic-related differences, as well as HF resultant from neglected tropical diseases such as Chagas disease.

A study conducted by Yusuf et al.^16^ revealed higher mortality rates from cardiovascular diseases in middle- and low-income countries when compared to high-income countries (3.99 events per 1,000 person-years versus 5.38 and 6.43 events per 1,000 person-years, respectively; p<0.001).^16,17^ This finding suggests that similar patterns may exist when comparing economically distinct regions within Brazil. Additionally, a strong association between a higher prevalence of uncontrolled risk factors, commonly observed in underdeveloped countries, and worse cardiovascular outcomes, including a higher incidence of HF, has already been demonstrated.^16,18^ Therefore, we can expect a similar relationship in the country.

Socioeconomic determinants significantly influence the characteristics and prognosis of HFrEF. However, it is important to note that available HF data that influence management strategies in Brazil are primarily derived from American and European registries, which may not accurately represent the HF population in developing countries. Moreover, randomized clinical trials carried out in specialized tertiary centers could potentially inflate the prevalence of specific etiologies.^14,19,20^ While ischemic heart disease is the primary cause of HF in developed countries, we anticipate that hypertension and Chagas disease will have a significant impact as causes of HF in Brazil. In addition, we expect that the adherence to guideline-directed medical therapy may be lower than that reported in European and American countries.^5,14,17^

To implement effective strategies for managing HF, it is essential to have a detailed understanding of how the disease presents itself. This will be a pioneer study conducting a multicenter investigation of patients with chronic HF across different Brazilian regions. The premise is that this information will enhance our comprehension of the disease's manifestation, comorbidities, and treatment patterns. By identifying regional disparities, we hope to develop new strategies to tackle the disease and support future public health policies tailored to the unique characteristics of each Brazilian region.^19-23^

This study has limitations. There might be some potential information bias arising from self-reported data. However, the study follows standardized protocols and is supported by continuous training efforts to minimize overall bias.

In conclusion, the Rosa dos Ventos Study aims to characterize regional variations in HF and assess the 12-month prognosis in Brazil. The findings will provide valuable insights into the variations in HF prevalence, management practices, and patient outcomes across different regions of the country. The results will serve as basis for targeted interventions and resource allocation, ensuring that regions with higher disparities receive the necessary support and resources to improve patient outcomes and reduce healthcare inequities. Moreover, a comprehensive registry study will likely help guiding the development of evidence-based guidelines and best practices that address the specific needs of different regions, fostering more equitable and tailored approaches to HF management nationwide.
